# Does Physical Exercise Modify the Pathophysiology of Alzheimer’s Disease in Older Persons?

**DOI:** 10.14283/jarlife.2024.11

**Published:** 2024-05-22

**Authors:** J. Raffin

**Affiliations:** 1. Institut Hospitalo-Universitaire (IHU) HealthAge, Toulouse, France;; 2. Institut du Vieillissement, Gérontopôle de Toulouse, Centre Hospitalo-Universitaire de Toulouse, 37 allées Jules Guesde, 31000 Toulouse, France

**Keywords:** Alzheimer’s disease, physical exercise, biomarkers, amyloid, tau

## Abstract

Physical exercise is well known for its benefits on brain health. However, the mechanisms through which these benefits occur remain discussed, especially in the context of cognitive conditions such as Alzheimer’s disease. The present short review summarizes the findings of interventional studies that examined the effects of exercise training on the specific and non-specific biomarkers of Alzheimer’s disease. Controlled exercise intervention studies published in the English language were selected if they assessed the effects of a physical exercise intervention of at least 2 weeks in middle-aged or older adults on one of the following biomarkers measured either in the brain, the cerebrospinal fluid or the blood: beta-amyloid, tau, neurofilament light chain, and glial fibrillary acidic protein. Overall, there was no strong evidence of significant effects of exercise interventions on any of the selected biomarkers. However, in specific populations, such as women with obesity, pre-diabetes, or depression, favorable changes in blood beta-amyloid concentrations were reported. Further benefits on cerebrospinal fluid beta-amyloid were also demonstrated in *APOE-ε4* allele carriers with Alzheimer’s disease. In conclusion, the current evidence suggests that physical exercise does not modulate the pathophysiology of Alzheimer’s disease in the overall population of middle-aged and older adults. Nonetheless, some specific populations, such as women with metabolic disorders and Alzheimer’s disease patients with *APOE-ε4* genotype, seem to be favorably affected. Further studies, including long follow-ups, large sample sizes, and concomitantly assessing the effects of other factors such as sedentary behavior and diet, are required to bring further evidence to the field.

## Introduction

**C**ognitive impairment and dementia are major causes of disability during aging (1). The current number of people living with dementia has been estimated at about 50 million, and this number will triple by 2050 (1, 2). Hence, preventing cognitive decline and dementia represents a major goal in aging societies, given the economic and social impact they induce (3). Alzheimer’s disease (AD) is the leading cause of dementia (4) and is notably characterized by an abnormal accumulation of dysfunctional beta-amyloid (Aβ) and phosphorylated tau proteins in the brain (5). In addition, the pathophysiology of AD also involves neuronal damages and neuro-inflammation that are respectively mirrored by an increased production of neurofilament light chain (NFL) and glial fibrillary acidic protein (GFAP) (6). Hence, all together, Aβ, phosphorylated tau, NFL and GFAP have been defined as main biomarkers for AD, the former two being core biomarkers and the latter two being non-specific biomarkers (6).

Various strategies to prevent AD have been developed, including drug therapy trials (7) as well as lifestyle interventions comprising cognitive stimulation, diet regulation, and physical exercise (8). While the positive effects of physical exercise on cognitive function are well demonstrated (9, 10), the pathway through which physical exercise induces beneficial effects remains unclear (11, 12). More specifically, the question of whether chronic physical exercise modulates the physiopathology of AD has not been established, and only a few studies have been conducted in humans (11). The present narrative review addresses this question by summarizing the effects of the published controlled interventions conducted in middle-aged and older adults that investigated the effect of regular physical exercise on the main biomarkers of AD (13).

## Methods

The present work is a short non-systematic narrative review on the effects of regular exercise on the main biomarkers of AD. We selected the interventional controlled studies that examined whether physical exercise intervention of any type (eg, aerobic, resistance, balance exercises, or multicomponent exercise training), conducted for at least 2 weeks, had an effect on AD biomarkers. AD biomarkers may have been measured either in the brain, the cerebrospinal fluid (CSF) or in the blood. Four biomarkers of AD were selected based on the latest version of the Revised Criteria for Diagnosis and Staging of Alzheimer’s Disease (6): Aβ (including Aβ38, Aβ40, Aβ42, and Aβ42/40 species), tau proteins (including total and phosphorylated species), NFL and GFAP. Only human studies conducted in adults were chosen with no restriction regarding age, sex, chronic diseases or cognitive status. Studies not published in the English language were not included.

## Results

Studies conducted on Aβ species, including middle-aged and older men and women with normal cognitive status, mild cognitive impairment or AD indicated no significant effect of exercise interventions, lasting from 8 weeks to 1 year and including 3 to 5 sessions of 45 to 60 min weekly, on either blood (14–18), CSF(19) or brain (20–22) amyloid levels, compared to control groups. However, subgroup analyses demonstrated that in *APOE-ε4* allele carriers with AD, an increase in CSF Aβ40 was observed in inactive controls after 16 weeks of follow-up while the carriers from the exercise group maintained their baseline concentrations (19). Other interventions specifically conducted in older women reported that 12 to 16 weeks of resistance exercise training induced a significant reduction in blood Aβ42 concentrations in obese (23) or pre-diabetic (24) individuals, compared to inactive groups. Such changes were accompanied by significant reductions in glycated haemoglobin (24). Likewise, 12 weeks of Taekwondo exercise administrated in older women with depression significantly reduced the blood levels of Aβ42 (25) compared to no exercise. Exercise interventions performed in older women with no specific health condition reported mixed findings with both significant changes and no change reported after 12 (26) and 16 weeks of aerobic training (27).

Regarding the effects of exercise on tau proteins, studies are scarce but it has been shown that in non-demented middle-aged individuals, 2 weeks of resistance exercise concomitant to a bed-rest intervention did not modulate the blood levels of total tau compared to bed rest alone (28). Likewise, 6 months of cycling exercise had no impact on the blood levels of phosphorylated tau 181 in cognitively healthy older adults (18). Furthermore, in older adults with AD, neither 16 (19) or 24 weeks (17) of aerobic exercise impacted the concentrations of total and phosphorylated tau proteins measured in the CSF or in the blood.

Similar negative findings have been reported on the non-specific markers of AD. In non-demented middle-aged and older adults, 2 weeks of resistance exercise conducted in parallel to a bed rest protocol did not impact NFL and GFAP blood concentrations compared to bed rest without exercise (28). Similarly, the blood concentrations in GFAP and NFL were not affected by 6 months of aerobic training in older adults with no cognitive impairment (18). A long-term intervention of 2 years of combined resistance and aerobic exercise reported no effects on blood concentrations in NFL in comparison to a control group (29). In patients with AD, it was reported that, compared to a no-exercise group, 16 weeks of aerobic training did not produce any significant changes in CSF NFL concentrations (30), even in subjects that were classified as amyloid positive (CSF concentrations Aβ42 < 550 pg/ml) (19).

## Discussion

Overall, the studies included in this short review demonstrated no effect of exercise interventions on the main biomarkers of AD, which is in line with previous reviews on the topic (31, 32). Nonetheless, some factors such as sex, *APOE* genotype or health status, seem to modify the effect of exercise training, as favourable and significant findings regarding Aβ levels were reported in women with obesity (23), pre-diabetes (24), or depression (25) and in *APOE-ε4* carriers individuals with AD (19). Hence, it is possible that exercise still has an effect on specific populations that display risks factors for AD such as women (33), *APOE-ε4* genotype carriers (34), individuals with metabolic disorders (35) or depression (36). Notably, the favourable effects found in AD patients with *APOE-ε4* genotype is also in accordance with previous work demonstrating that *APOE-ε4* carriers display greater improvements in cognitive functions in response to exercise compared to non-carriers (37). Regarding the lack of effects in the overall population, because most of trials were performed in older adults, it is possible that an advanced age might counteract the benefits of physical training, although some authors have reported greater effects of exercise on cognition in healthy adults older than 60 (38) compared to younger counterparts. Our findings also diverge from the observational studies and meta-analyses that demonstrated beneficial associations between PA and amyloid (39, 40), NFL (41–43) and GFAP (43) levels, although the relationships with tau remain contrasted (39, 44–48).

Even though interventional studies reported little effects on the specific biomarkers, PA may still improve cognition through other pathways. Recent reviews published on this topic have reported that chronic exercise has a positive effect on brain glucose metabolism, vascular function, and BDNF concentrations, along with providing benefits on cognition (11, 32, 38). This indicates a pleiotropic effect of exercise that is consistent with its positive impact on the biological hallmarks of aging (49), which are thought to be the common roots of most of age-related diseases (50). Future studies should thus focus on long term interventions not only in middle-aged and older adults, but also in young adults as abnormal proteins deposition may start decades before the disease onset (51, 52). Studies examining the factors that may moderate the effect of exercise, such as sex, genotype, or health status are also required, as well as studies with large sample sizes, given that the subject samples of the studies selected for this review were relatively small, ranging from 156 to 14 individuals. We also recommend that cognition should be assessed along with the measurement of the neurodegeneration biomarkers in order to determine whether changes in the latter could mediate changes in the former. In addition, most of the studies included herein focused on tau, amyloid, and NFL proteins, but there is a lack of evidence regarding the impact of exercise on GFAP. Yet, the research on the non-specific markers of AD, namely NFL and GFAP, remains important. Indeed, GFAP is an indicator of astrocyte activation (6) and has been shown to be an early predictor of Aβ production (53) while NFL reflect axonal damages (6). Yet, higher blood levels of both NFL and GFAP have been associated with reduced cognitive capacity (54, 55) and greater prospective cognitive decline (54). Importantly also, the biomarkers examined herein may interact with each other, such that the benefits of exercise on one biomarker may depend on the levels of other biomarkers. Interventional studies simultaneously measuring the effects of several biomarkers and examining their interactions may thus provide significant contributions to the field.

In conclusion, physical exercise interventions do not demonstrate favorable AD-modifying effects, except in women with impaired metabolic health or depression and *APOE-ε4* carriers patients with AD. While more studies are needed given the paucity of available evidence, other important factors such as diet (56, 57), cognitive stimulation (58), or sleep quality (59) may also modulate AD pathophysiology and should be explored collectively. Importantly, sedentary behaviour, which demonstrated significant association with incident dementia (60), may have deleterious independent and/or exercise-counteracting effects on brain physiopathology. All of these factors may act synergistically and potentiate the single effects of each individually, emphasizing the need for holistic approach interventions to prevent dementia (61).
